# Organic Brake Friction Composite Materials: Impact of Mixing Duration on Microstructure, Properties, Tribological Behavior and Wear Resistance

**DOI:** 10.3390/polym14091692

**Published:** 2022-04-21

**Authors:** Fatma Makni, Anne-Lise Cristol, Riadh Elleuch, Yannick Desplanques

**Affiliations:** 1 Laboratory of Electro-Mechanical Systems (LASEM), National School of Engineers of Sfax (ENIS), University of Sfax, Sfax 3029, Tunisia; riadh.elleuch@gnet.tn; 2 Univ. Lille, CNRS, Centrale Lille, UMR 9013-LaMcube-Laboratoire de Mécanique, Multiphysique Multi-échelle, F-59000 Lille, France; anne-lise.cristol@centralelille.fr

**Keywords:** brake friction materials, mixing duration, properties, tribological behavior, wear

## Abstract

The lack of knowledge on the link between the manufacturing process and performance constitutes a major issue in brake lining development. The manufacturing process of organic brake friction composite materials includes several steps (mixing, preforming, hot molding and post-curing), which define their final microstructure, properties and performances. This study focuses on the effect of mixing duration on the microstructure, properties and tribological behavior of organic friction composite materials. The adopted methodology is based on simplified formulations effective in limiting synergistic effects by reducing the number and size distribution of constituents. Two simplified materials are here developed according to the mixing duration of the constituent introduction sequence. The microstructural characteristics are studied using 2D and 3D analyses, and then correlated with the thermophysical and mechanical properties. Wear mechanisms and tribological behavior are studied in relation to the microstructure and properties of the materials. The results show the effect of mixing duration as regards particle distribution and fiber arrangement. The distribution and size of fiber entanglements contribute to the formation of carbonaceous particle clusters, which create bulk bridges improving thermal conductivity. Moreover, the arrangement of rock fibers affects density, porosity and thermo-physical properties. In addition, the mixing disrupts the cohesion of fiber bundles with the matrix, affecting compressive modulus and wear behavior. This microstructural defect also fosters abundant third-body source flow, which disturbs the tribological circuit and behavior. Porosities induced by fiber entanglements, having a large and irregular size and distribution on the frictional surface, result in low wear resistance and alter the frictional stability.

## 1. Introduction

Friction materials represent important parts of brake systems extensively used in the automotive, railway, and other transport fields [1,2]. These brake friction materials have complex formulations that should fulfill several performance requirements, such as stable friction coefficient, low wear, low fade, less brake vibration and noise, and environmental friendliness [3,4]. These requirements are affected by the formulation, the selection of the constituents, and the setting of manufacturing parameters. Several studies have focused on the optimization of friction material manufacturing processes [5,6,7,8]. For example, Ertan and Yavuz [9] investigated the effects of the manufacturing parameters on the tribological behavior of friction materials in order to achieve optimal manufacturing parameters and thus improve their performances. Ficici et al. [10] used the Taguchi analytical methodology to explore the impact of manufacturing on the tribological behavior of brake friction materials. The impact of the types and relative amounts of raw material constituents on the friction material’s properties and performances have also been widely studied [11,12].

A composite friction material is essentially a multi-constituent material that is highly heterogeneous. Pai et al. [13] revealed that using waste tire rubber particulates (WTRPs) as the micro-fillers in brake lining materials led to improved coefficients of friction and wear resistance. Studies on the relationship between the constituent arrangement and distribution induced by the manufacturing process and the resulting microstructure, properties and performances of brake friction materials have not been carried out yet.

Furthermore, little research has focused on the impact of the manufacturing process on the resulting microstructure [14,15]. Indeed, Makni et al. [15] investigated constituent repartition induced by mixing organic friction composite materials, by means of image analysis. However, previous studies [16,17] provided only 2D microstructural analyses, which remain insufficiently representative of constituent distribution in the material’s volume. Besides this, these works have not provided sufficient information about synergies between constituents, which have a major impact on the properties and performances of organic brake friction materials. Consequently, the elaboration–microstructure–properties–performances continuum is still not well established for these multi-constituent materials. In fact, microstructural characteristics, namely, the arrangement of fibers, the distribution of constituents, and porosities induced by the manufacturing process are still not well investigated. The available data are not very detailed and concern only average fiber fractions and void contents, with some representative microstructural analyses [14,16,17]. 

As we have mentioned, there has been some interest on the impact of the manufacturing process, namely, hot molding and post-curing, on friction materials’ properties and performances [16,17]. However, the mixing step is still not well investigated. Mixing is the first step in brake pad production. This operation depends on several factors: the order in which ingredients are added, mixing duration, speed, and loading capacity. Each parameter influences the quality of the mixture, which defines the microstructure and properties of the friction material. The impact of the step parameters on the microstructural, thermo-physical and mechanical properties, as well as the tribological behavior, of friction materials remains poorly understood. Indeed, no correlation has been revealed between the resulting microstructure, properties and performances of brake materials [18]. Therefore, the mixing step should be investigated in a thorough way.

This leads us to the aim of this paper, which is to study the impact of mixing duration on the resulting microstructure, properties and tribological behavior of friction materials. Besides this, this work helps us to understand the elaboration–microstructure–properties–performance continuum through a systemic, multi-scale and multiphysical analysis. Indeed, the composite material is considered as a system of interacting components (matrix, particles, and fibers). The properties and performances of the material are apprehended as the result of these interactions, which allows us to explain the elaboration–microstructure–properties–performances continuum. To achieve these objectives, an experimental methodology based on material simplification is adopted. Thorough microstructural analyses using 2D and 3D characterizations are performed, and then correlated with the resulting properties (thermo physical and mechanical properties) and tribological and wear behavior of the studied materials.

## 2. Materials and Methods

### 2.1. Simplified Materials

The adopted simplification methodology is based on the reduction of constituent number and size distribution, while retaining their efficiency in braking situations [17,19]. The aim of this approach is to limit synergistic effects and facilitate the identification of each component of the microstructure, and thus to access the friction materials’ properties and performances. Six constituents were retained (phenolic resin, calcium carbonate, rubber, graphite, alumina and rock fibers) with reduced size distributions, as shown in Table 1. Resin, calcium carbonate and alumina present very fine particles (mean diameter < 6 µm) and constitute the matrix of these composite materials. A reduction in the size distribution of constituents was performed using the sieving method [20]. The elaboration process of brake friction materials includes several steps (mixing, cold preforming, hot molding, post-curing). In this study, the impact of the mixing step on the resulting microstructure and properties of friction materials was investigated. The order in which ingredients are added (introduction sequence) and mixing duration were defined according to the results discussed in a previous work [15]. The latter suggested an order and duration of the constituent introduction sequence, which would permit us to obtain a uniform distribution of the particles. Five introduction sequences of simplified formulation constituents have been defined, as given in Table 2. In this study, two materials were elaborated depending on the mixing duration of each constituent introduction sequence. The simplified materials were elaborated using a laboratory mixer, which reproduces the same mixing mechanisms as an industrial one. The constituents of the two materials were incorporated into the mixture in same defined order. The first material, referred to as M1, was created using a defined introduction sequence of constituents, with a total mixing duration of 706 s. The second, M2, was elaborated with the same introduction sequences, but with a very short mixing duration of 40 s (Table 2). This short duration was fixed to ensure the sufficient brazing and reasonable repartition of the constituents. Before elaborating the mixture of M2, the components, namely, graphite and rubber particles, were pre-coated with matrix particles to guarantee the cohesion of the final material [21]. The adopted methodology aims to establish a link between the microstructural characteristics induced by the mixing step, and the properties and tribological behavior of the composite friction materials.

After mixing, a series steps was carried out: first, cold preforming, then hot molding at a curing temperature of 140 °C for 11 min under a pressure of 200 bars, and finally post-curing at 160 °C for 8 h. At the end of the manufacturing process, several finishing operations were performed to derive a flat plate of 16 mm thickness and 400 × 400 mm width. For this work, the specimens sampled from the resulting plates were cylindrical, with a parallel axes to the normal direction of the manufactured plate. This sampling direction enables the analysis of the transverse isotropy of the microstructure and the properties of these materials [17]. The normal direction corresponds to the direction of compression during the preforming and hot molding steps, which presents the direction of normal load application on a brake lining when in use.

### 2.2. Microstructure Characterizations

#### 2.2.1. 2D Microstructural Analysis

In the first step, the characterization of the microstructure was carried out by means of Scanning Electron Microscopy (SEM) using the following variables: an accelerating voltage of 15 kV and a distance between the sample and the electron gun (working distance) of 35 mm. The selected mode of electron detection was backscattered electrons (BSE), used to distinguish the different constituents and their distributions on the studied materials’ surfaces via image contrast. The specimens used for microstructural examination were polished following standard procedures. The aim of this investigation is to provide information about the constituent distribution and microstructural characteristics of the resulting materials.

#### 2.2.2. 3D Microstructural Analysis

The size distribution, arrangement and spatial repartition of the constituents of the two materials M1 and M2 were analyzed by 3D X-ray tomography using laboratory equipment with a procedure already described in detail in Makni et al. [20]. Cylindrical specimens with a length of 16 mm and a radius of 6 mm were sampled from each material in the normal direction (Z direction) and scanned using an X-ray microtomographic apparatus (Ultratom, RxSolution©). The transmission X-ray tube (W target) was operated at a voltage of 160 kV and an intensity of 275 μA. The voxel size was set at 4.4 μm^3^. Reconstructed images were analyzed using the Image J 1.46r software of the Wayne Rasband National Institutes of Health USA (http://imagej.nih.gov/ij (accessed on 19 April 2016)). This technique allows one to evaluate particle distribution and arrangements in the material volume, and mark internal defects and constituents of interest [22]. To assess the constituent spatial distribution in the studied material’s volume, 3D analyses were carried out on specimens with a radius of 16 mm and length of 16 mm.

### 2.3. Thermo-Physical Characterization

The physical (density and porosity), thermal (thermal conductivity, specific heat capacity and thermal effusivity) and thermally induced (thermal expansion) properties of M1 and M2 were studied. The density was measured using Archimedes’ principle. The specimens were weighed in air then in water using a Mettler Toledo electronic balance with 0.5 g accuracy. Theoretical porosity was calculated using the following expression:

(1) $$Porosity\left(\%\right)=1-intensificationrate\left(\%\right)$$

(2) $$Intensificationrate\left(\%\right)=\frac{\rho_{m}}{\sum \frac{m_{i}x\rho_{i}}{100}}\times 100$$

where *ρ_m_* represents the bulk specific density (g/cm^3^), *m_i_* corresponds to the weight (%) of each ingredient of the formulation and *ρ_i_* represents the absolute density of each ingredient of the formulation (g/cm^3^). Thermal expansion $\left(\alpha \right)$, thermal conductivity (λ) and specific heat capacity $\left(Cp\right)$ measurements were performed according to the defined parameters and using specific equipment detailed in a previous study [20]. The thermal effusivity (ε) was calculated using the measured values of conductivity, density and specific heat capacity, as follows:

(3) $$\varepsilon =\sqrt{\rho \lambda Cp}$$

The results of these characterizations are reported in Table 3. Three samples of each material were tested, and average values of the measures are given with a standard deviation value.

### 2.4. Mechanical Test

**Figure 1:**
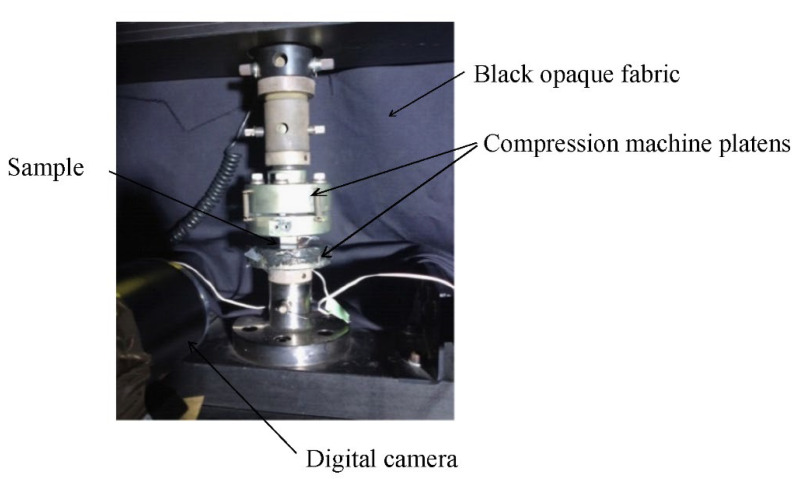
Compression test set-up.

Simplified materials were characterized in terms of their mechanical properties using a compression test. The latter was performed on an Instron electromechanical machine with a load capacity of 10 kN. The specimens were parallelipedic with a width of 20 mm and a height of 16 mm. The deviation in the parallelism of the top and bottom faces, in contact with the non-rotated platens, was less than 20 µm. Both contact faces of the sample were lubricated with graphite to promote sample–plate sliding and limit its barrel deformation. A speckle was applied on one side face with an airbrush [21] and the digital image acquisition was performed with a Ximea camera at a frequency of 5 frames/s. In order to derive a uniform illumination of the speckles of the studied face, a projector was positioned at a distance of 4 m, and a black cloth masked the organs of the machine to reduce the light reflections on the sample (Figure 1). The samples were machined in the normal direction to the plane of the brake friction materials. Three stress levels of 2 MPa, 5 MPa and 10 MPa were applied to the samples. Each stress level was applied five times. The speed of plateau displacement was set at 0.01 mm/s [20].

The Digital Image Correlation (DIC) method was employed to identify strain/stress localizations [23]. To achieve the post-treatment of all results, Digital Image Correlation (platform yadics, http://yadics.univ-lille1.fr (accessed on 19 April 2016)) was used. The compressive modulus was estimated using the measurement of strain fields (Table 4) [24]. 

### 2.5. Evaluation of Tribological Behavior

#### 2.5.1. Tribometer Description

The evaluation of the tribological behavior was performed by means of a wear test. The latter was carried out on a pin-on-disc tribometer with plane-to-plane contact (Figure 1). This apparatus was composed of a spindle, at the end of which was mounted a test disc, driven in rotation by a brushless electric motor, which guaranteed a high degree of regularity of rotation, along with a load application and force measurement device. This device consisted of a clamp with a diameter of 16 mm, in which the friction material sample was fixed, mounted on a slider driven by an elastic clearance free connection for the load application. The load was applied by an elastic system of stiffness 32.7 N/mm, which involved the compression of a helical spring. The normal and tangential forces were measured by two strain–gauge sensors implemented on the test cell. These measurements have been used to calculate the friction coefficient.

#### 2.5.2. Experimental Protocol

**Figure 2:**
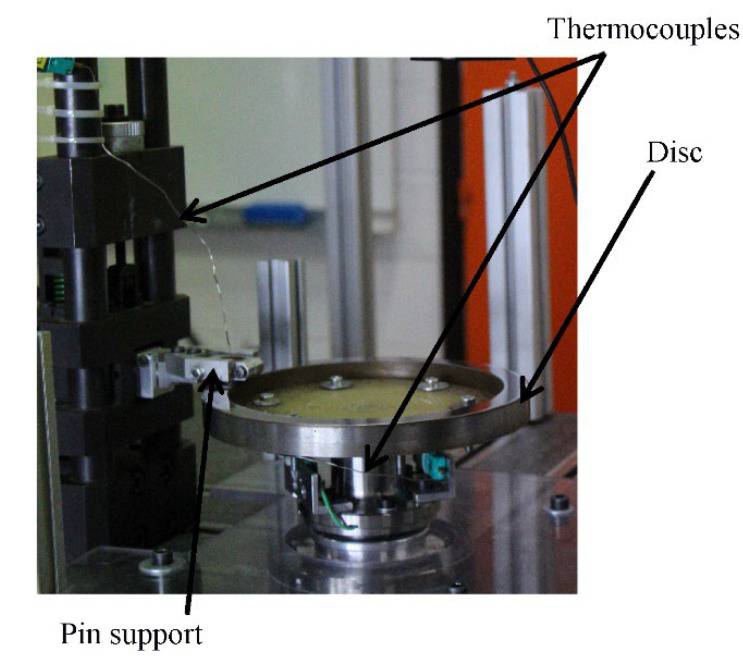
Pin-on-disc tribometer.

The friction tests were carried out with a pin made of the friction material rubbing against a gray cast iron disc (Figure 2). The pin had a cylindrical shape with a diameter of 16 mm and a height of 16 mm, and the disc track had an average friction radius of 100 mm and a thickness of 20 mm. The disc was made of lamellar graphite cast iron (ENGJL 250). The pin’s surface was rectified, and the surface of the disc was polished to grade 500 rpm, and then 1200 rpm, at increments of 200 rpm [17]. The out-of-plane runout of the disc friction track was controlled so that it was limited to a few tens of micrometers. Tribological tests were performed under low-energy conditions at a rotating speed of 600 tr/min, i.e., a sliding speed of 6.3 m/s on the mean track radius, and an applied load of 100 N.

The friction tests were interrupted at different time points, while taking care not to exceed a 100 °C disc temperature in order to correlate the evolution of the friction coefficient with the tribological mechanisms and phenomena induced during the friction. 

The retained duration was 5 min. For each material, five sequences of run-in followed up by a sliding test of 5 min were performed. Disc and pin temperatures were measured by means of the K-type thermocouples located on the average friction radius, 2 mm and 3 mm below the friction surface, respectively [19,25]. The test conditions led to a low heating of the disc and the pin, limiting the temperature rise to a few tens of degrees Celsius at the surfaces.

#### 2.5.3. Evaluation of Wear Behavior

The friction tests were carried out on the same tribometer, with the same set-up, the same sample geometry, and the same grey cast iron disc, so as to preserve the same influence of the tribological system on the friction and the same wear behavior of the M1 and M2 materials. The formulation of these materials was chosen with an identical and limited quantity of fine abrasive particles to limit the wear of the disc, which we attempted to keep be as neutral as possible in this study. Under these conditions, the wear of the disc results in very fine particles of iron oxides, which contribute to the formation of a layer of third bodies protecting the first bodies. The formation of this third body layer has been studied in detail [26], with tests carried out on the same tribometer, with very similar experimental conditions (900 rpm, 230 N). The materials considered were a grey cast iron disc and an organic composite friction material, with a more aggressive formulation regarding abrasive wear than that of materials M1 and M2. The study shows the formation of a third body layer, about ten micrometers thick, which separated the first bodies. This nanostructured layer consisted of a mixture mainly composed of very fine magnetite particles (diameter approximately 10 nm), provided by the wear of the disc, comprising mixed particles produced by the wear of the organic composite friction material. Desplanques et al. [27,28] have shown that the formation of such iron oxide layers between the first two bodies can help in reducing wear. In our case, disc wear is particularly limited due to the low abrasive particle content of both M1 and M2 materials. Under these conditions, and due to the very large surface area of the disc friction track compared to the friction surface of the pin, the thickness loss due to disc wear remains very small—sub-micrometric—compared to the micrometric thickness loss of the pin [17,25].

The wear mechanisms induced by friction were investigated using SEM observations and EDS analyses of the rubbed surfaces. The EDS analysis of the chemical composition of the flat plates at different points for each material was performed to reveal the wear behavior of the studied materials. The results of this analysis are listed in Table 5. To estimate the wear rate, the pin thickness was measured before and after each friction test (Table 6).

## 3. Results and Discussion

### 3.1. Microstructure

**Figure 3:**
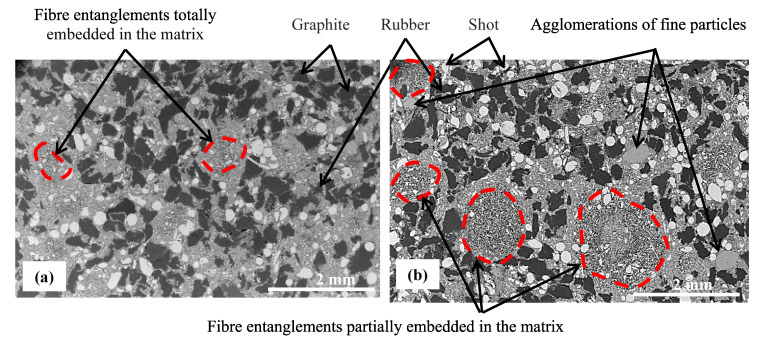
Microstructure of materials (**a**) M1 and (**b**) M2 (SEM-BSE).

**Figure 4:**
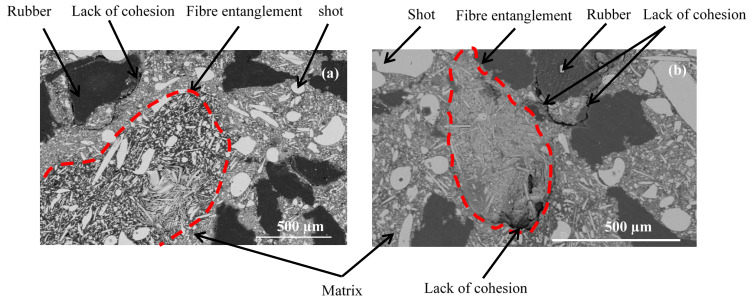
Microstructural defects in materials (**a**) M1 and (**b**) M2 (SEM-BSE).

Figure 3 illustrates the different components of the two materials: fine particles (resin, calcium carbonate and alumina) constitute the matrix, which contains the other constituents (rubber, graphite, rock fibers (shots and fibers)). Micrographs of M2 display more numerous fiber entanglements of rock fibers, which present bigger and more variable sizes than those of M1 (Figure 3b). Moreover, they seem to be less embedded in the matrix. The fibers appear to have mostly emerged from the matrix in M2. SEM micrography of M2 (Figure 4b) provides evidence of the poor interface adhesion between the rock fibers and the matrix. The morphologies of the fiber bundles in the two materials seem to be similar, since they result from the re-entanglement of fibers during the mixing step [20].

Several agglomerated particles have been revealed in M2 (Figure 3b). These agglomerations correspond to matrix particles, which indicates that they were not well dispersed in this material. Figure 4 shows a lack of cohesion between rubber particles and the matrix, which seems to be more pronounced in M2. 

#### 3.1.1. Spatial Constituent Distribution

**Figure 5:**
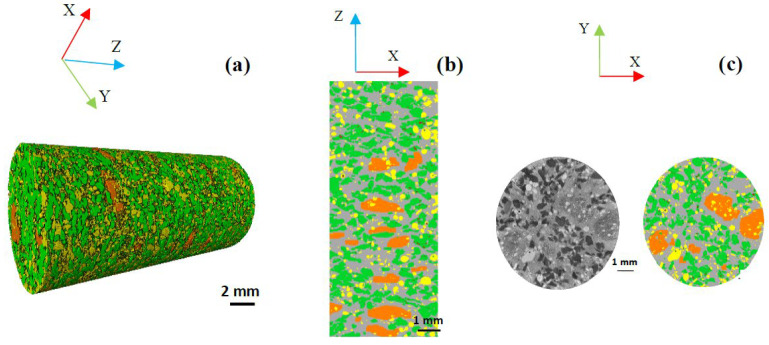
Spatial constituent distribution in M1; (**a**) 3D visualization, (**b**) 2D longitudinal visualization in XZ plane, (**c**) 2D orthogonal visualization in XY plane. Fiber bundles (orange), carbonaceous particles (green), matrix (gray), shots (yellow), voids (red).

**Figure 6:**
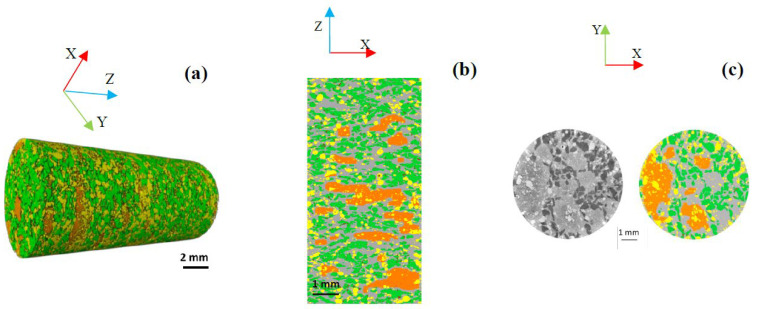
Spatial constituent distribution in M2; (**a**) 3D visualization, (**b**) 2D longitudinal visualization in XZ plane, (**c**) 2D orthogonal visualization in XY plane. Fiber bundles (orange), carbonaceous particles (green), matrix (gray), shots (yellow), voids (red).

It should be mentioned that, in all the 2D and 3D visualizations given by Figure 5 and Figure 6, the fiber entanglements and carbonaceous particles are marked in orange and green, respectively. The shots and matrix are highlighted in yellow and gray, respectively. The 2D orthogonal and longitudinal visualizations are displayed in the XY and XZ planes, respectively. 

The 3D visualizations shown in Figure 5 and Figure 6 represent the spatial distributions of constituents in both materials. Figure 6 reveals the presence of several rock fiber entanglements in the volume of the M2, which are more numerous and bigger than those in M1. These entanglements’ shapes are irregular, and their sizes are variable, as in M1. The longitudinal sections in Figure 6b show that fiber entanglements are randomly dispersed throughout the height of the sample, and their size can exceed 3 mm. As for the carbonaceous particles, they most often appear grouped and interconnected in different sections of M2. In M1, the inter-particle distances between carbonaceous particles are more important, and their interfaces with the matrix are more numerous. 

**Figure 7:**
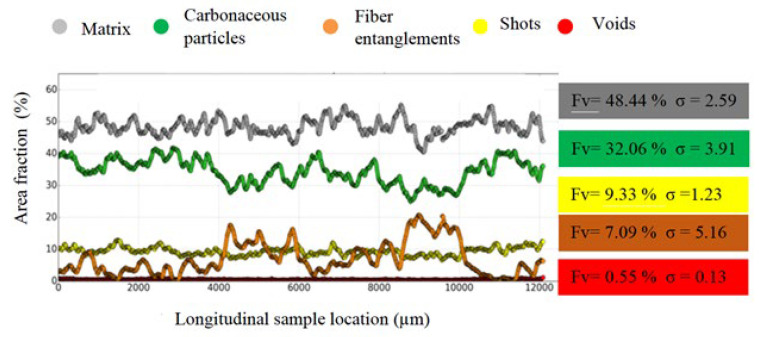
Spatial constituents’ distributions according to the longitudinal location (Z-axis) along the sample in M1, with the volume fraction (Fv) and standard deviation (σ) of each constituent.

**Figure 8:**
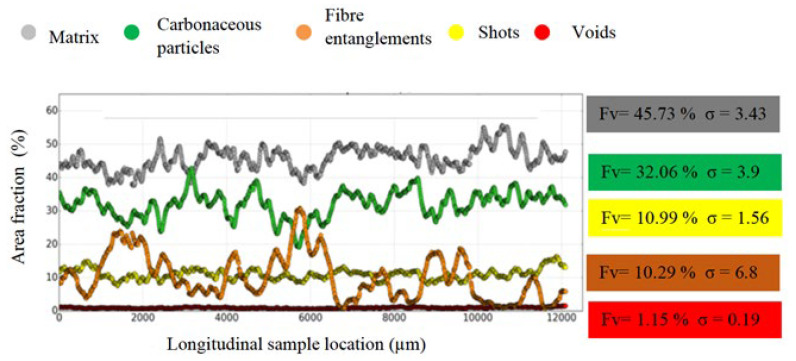
Spatial constituents’ distributions according to the longitudinal location (Z-axis) along the sample in M2, with volume fraction (Fv) and standard deviation (σ) of each constituent.

These observations are confirmed by Figure 7. This figure presents the spatial distribution of components using the area fraction of each component relative to the entire cross-section area of XY, versus the longitudinal sample location of M1 [20]. The fluctuations in the components’ spatial distributions in M1, and especially for the fiber entanglements and matrix, appear to be more important than those revealed for M2. This is confirmed by the standard deviations (σ) of the fiber entanglements and matrix, which are 6.8 and 3.43 for M2, compared to 5.16 and 2.59 for M1, respectively (Figure 7 and Figure 8).

The fiber entanglement volume fraction (Fv) calculated for M2 (10.29%), given is Figure 7, is higher than that obtained for M1 (7.09%), consistent with the poor fiber dispersion due to the short mixing time. It can be noted that the void volume fraction of 1.15% is double the void volume fraction of M1 (0.55%). The greater presence of these voids is consistent with the presence cohesive defects at the constituent interfaces with the matrix, revealed by the microstructural analysis. These results agree with the 2D analyses, whereby the reduced mixing time resulted in a very heterogeneous microstructure. Although the constituents were pre-coated with resin, their cohesion with the matrix at the interfaces is slightly worse than that seen in M1.

**Figure 9:**
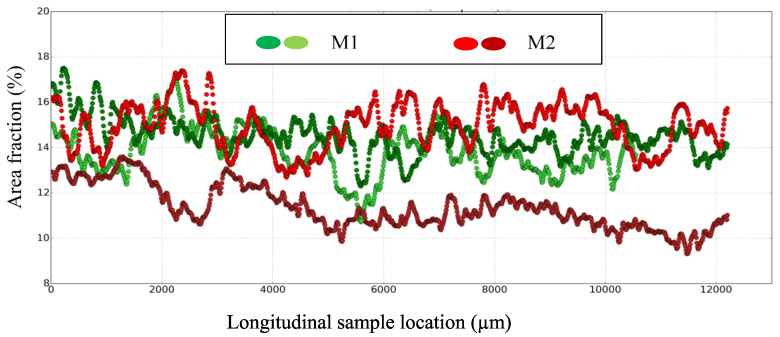
Spatial carbonaceous particles in two 16 mm-diameter specimens sampled from M1 and M2.

Figure 9 shows the spatial distribution of carbonaceous particles in the two M2 samples, compared to that obtained in the two M1 samples. It can be noticed that the area fractions obtained for the two M2 samples do not overlap, which means that the sizes of the samples are insufficient to be representative of the distribution of carbonaceous particles. This confirms the very high heterogeneity of the microstructure of M2, which is mainly induced by the random distribution and the very large size of the fiber entanglements.

### 3.2. Thermo-Physical Properties

The results of the thermo-physical characterizations of the two studied materials are given in Table 3. They show a higher density and a lower porosity for M1 compared to M2. This can be explained by the presence of voids induced by the lack of cohesion between the matrix/fiber entanglement interfaces in M2. 

These results are in agreement with Maleque and Atiqah [29], who found that the material porosity decreases with increases in the density value due to the presence of voids. Regarding the thermal and thermally induced properties, thermal conductivity and thermal expansion show significant differences between the two materials, while thermal effusivity and heat capacity are comparable in both. As the microstructural analysis has shown, the rock fibers in M2 present less cohesion with the matrix compared to M1. In fact, the fibers in M2 are mostly found to be partially embedded in the matrix.

This presents an important factor that impacts thermal expansion. Indeed, high cohesion allows the rock fibers to block the thermal expansion, while low cohesion leads fibers to slip in the matrix during its thermal expansion, thus facilitating the thermal expansion. This may justify the lower coefficient of thermal expansion seen in M1.

The microstructure of M2, which presents higher thermal conductivity, is strongly influenced by entanglements, with microstructural heterogeneity becoming more pronounced the larger and more numerous the entanglements are. In this material, the distribution of carbonaceous particles, namely, graphite (heat-conducting particles), constrained in small inter-entanglement spaces, forms dense networks where the inter-particle spaces are reduced, which enhances thermal conductivity. This can explain the higher thermal conductivity of M2. These results agree with the work of [15,30,31]. These authors confirm that the distribution of constituents, especially fibers, has an influence on the thermal properties of composite materials.

### 3.3. Mechanical Properties 

**Figure 10:**
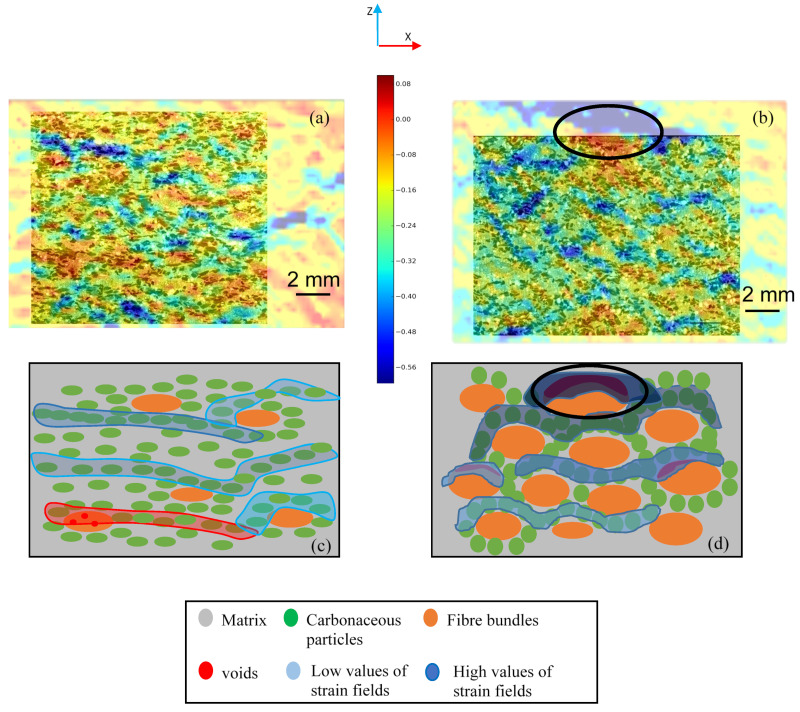
Strain fields Ezz (%) in the compression direction at 10 MPa superimposed on the microstructure for (**a**) M1 and (**b**) M2, and the schematic relationship with constituent distribution for (**c**) M1 and (**d**) M2.

Strain fields of 10 MPa, given in the compression direction (Ezz (%)), are presented in Figure 10. This latter shows that strain localizations correspond to the voids induced by the fiber entanglements, as well as to the rubber and graphite particles, which are not very stiff in comparison with the other constituents [21]. These fiber entanglements may contain porosities. In M2, bigger fiber bundles become more deformed with a higher amplitude (in darker blue) (Figure 10b,d). 

Besides this, Figure 10b,d reveals the high degree of amplitude deformation localization at the large surface entanglement of the M2 sample (circled in black), which may present voids and a lack of cohesion at the interfaces with the matrix. The load transfer at these weakly cohesive interfaces is a factor in heterogeneous strain accumulation [32]. Low strain amplitude localizations (in light blue) are observed at some entanglements of M1 and M2, which could be attributed to porosities. In M1, only a few entanglements undergo weak compression (in red and light blue) (Figure 10a,c). High strain–amplitude localizations (in darker blue), oriented perpendicular to the loading direction, correspond to the clustering of rubber and graphite particles (in green). These localizations are stronger and more developed (shown in darker blue) in the case of M2, as the density of carbonaceous particles in these clusters is higher for this material. 

Table 4 shows that compressive modulus values increase with stress levels. This may be explained by the presence of porosities in these materials [17,20]. Indeed, at low loads (2 MPa), voids that are slightly compressed close partially. As the load increases, the compression closes more voids, which leads to an increase in stiffness [25]. 

As regards the moduli obtained for M2, Table 4 reveals that they are lower compared to the compressive moduli of M1. According to several studies [33,34], the mechanical properties of fiber-reinforced composites depend on the length, orientation and distribution of the fibers in the material [29]. Besides this, the mechanical properties are related to the shear strength of the interface between the fibers and the matrix. In fact, the structural integrity of the composite material, and the ability of the interphase to transmit the load from the matrix to the embedded fibers, is ensured by the shear strength of the fiber–matrix interface. This may explain the lower compressive moduli revealed for M2, which display a pronounced lack of cohesion between rock fibers and matrix. These results are in accordance with [35,36], where the authors have shown that the strength of the composite materials depends on the properties of the reinforcing fibers and the quality of the fiber–matrix interfacial adhesion strength. Indeed, they revealed that the applied load can be transferred from the matrix to fiber more efficiently when the fiber–matrix adhesion strength is well established. In other words, strong fiber–matrix cohesion enables the better mechanical behavior of composite materials [37,38,39]. Microstructural analyses have shown the better bonding of the interface between the fibers and the matrix for M1. This justifies the higher compressive modules found for M1. Furthermore, the higher porosity revealed in M2 in Section 3.1.1 can explain its lower compressive moduli [40].

### 3.4. Tribological Behavior Analysis

#### 3.4.1. Friction Behavior

**Figure 11:**
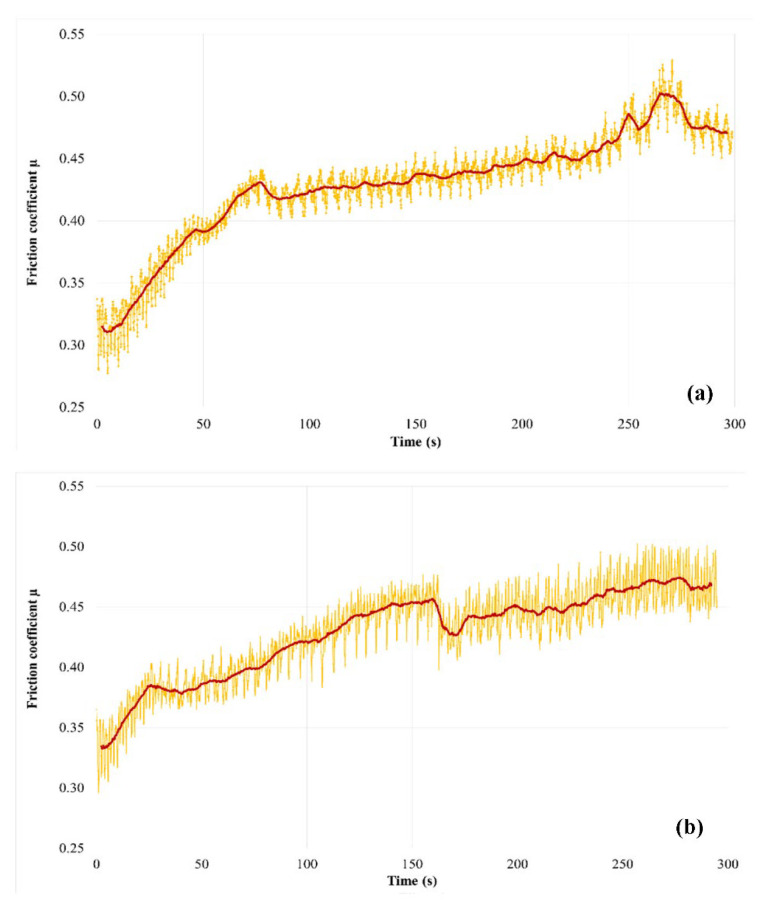
Evolution of the friction coefficient (µ) during the friction test for (**a**) M1 and (**b**) M2 (the average value of µ is obtained from a low pass filtering of the force measurements).

Figure 11 shows the time evolution of the friction coefficient (µ) obtained from the normal and tangential force measurements. The average value of µ was calculated using the low-pass filtering of the measurements. Figure 11a shows that the friction coefficient’s evolution in the case of M1 increases continuously during the test, in a more or less regular way. Two stages can be observed:

- A transition stage of about 100 s at the beginning of the test, characterized by a marked and irregular increase in the friction coefficient (whose average value increases from 0.31 to 0.42), which can be associated with the establishment of a sliding contact;
- A second stage characterized, by a smoother and more regular increase in the friction coefficient (from 0.42 to 0.47), disturbed by one-off increases in friction a little before the end of the test. We note that fluctuations in the time friction evolution show a fairly regular periodicity when friction evolves slightly, while they are more intense and disturbed at the beginning of the test, as well as during the final friction perturbations.

Figure 11b shows the friction coefficient evolution of M2. The transient phase of contact establishment is more difficult to distinguish. It extends to the whole first half of the test, where a sudden disturbance appears. During this period, the friction coefficient increases from 0.33 to 0.45. As in the case of M1, the second part of the test is marked by a gentler increase in friction (from 0.44 to 0.47 for the average value). Temporal fluctuations in friction of greater amplitude than in the case of M1 are noted, in which it is more difficult to distinguish a clear periodicity.

**Figure 12:**
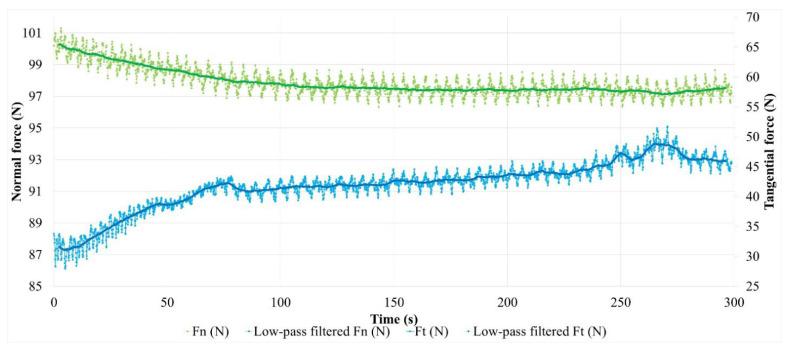
Evolution of contact forces for material M1.

Friction variations are indicative of changes at the interface in the mechanisms of load-bearing accommodation, sliding velocity and energy dissipation, which result from the evolution of the third body layer and associated material flows. To better understand these evolutions, the normal and tangential forces measured during the tests are analyzed in detail. Their evolution is presented in Figure 12 for M1.

**Figure 13:**
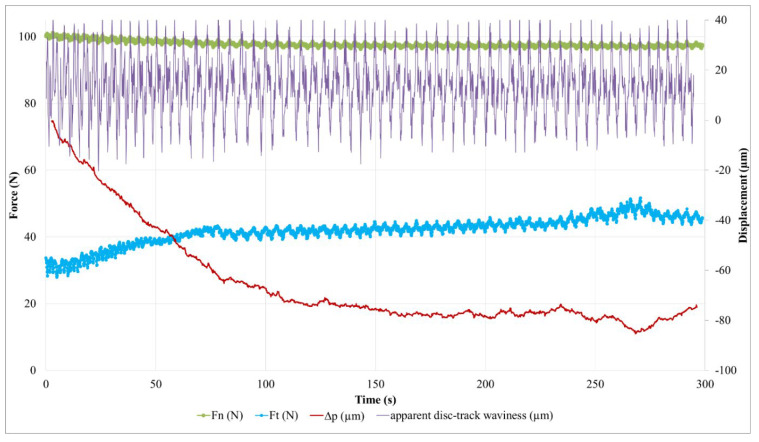
Evolution of contact forces, pin displacement (∆p) and apparent disc track waviness during the friction test for material M1.

Let us first consider the normal force in the case of the test on M1. The evolution presents, on the one hand, a regular, almost periodic fluctuation throughout the test (studied below), and, on the other hand, a decrease at the beginning of the test, as shown by the average value. This decrease is 2.4 N during the first 120 s. We should recall that the normal load is derived from the compression of an elastic system, mainly constituted by a helical spring. The stiffness of this system is 32.7 N/mm, measured under a 100 N load. It includes the stiffness of the strain gauge force measurement device. Thus, the 2.4 N drop in normal force is associated with a 74 µm relaxation of the device. This value corresponds to the pin displacement relative to the tribometer frame in the direction normal to the contact. Among the displacement sources, a deflection variation or a deformation under the load of the disc or the pin is excluded. This deformation is sub-micrometric, considering the involved rigidities under the load and the very low thermal expansions, and the increases in bulk temperature are less than ten degrees Celsius in the disc, the same as in the pin [16,17]. Moreover, pin displacement can only be partially attributed to the material losses of the first bodies: on the one hand, the pin thickness loss is about 5 µm for these tests while that of the disc friction track is imperceptible on this scale. On the other hand, the value of the normal force evolves slightly from one test to the next, without any adjustment of the compression setting of the loading system. The pin displacement is therefore reversible from one test to the next, the source of which is in the evolution of the plane to plane interface of the pin–disc contact. In Figure 13, the pin displacement curve is a translation of the filtered normal force curve, taking into account the loading system’s stiffness.

It should be noted that the reduction in the normal force is concomitant with a rapid and irregular increase in the tangential force (Figure 12). These phenomena are attributed to the progressive setting up of contact during the beginning of the sliding. Indeed, the “plane-to-plane” pin–disc compliance, lost at the beginning of contact, must be reconstituted at its end. The latter is established by a reconstitution of the third body-bearing plateaus and their redistribution by third body flow and compaction in the interface, taking into account the third body flow rates and the trapping capacity of the contact. Thus, during this transient stage, the drop in normal force reflects the progressive accommodation of the bearing in the contact by the third body redistribution, until a satisfactory contact compliance is achieved. This period of contact accommodation results in an increase in the friction.

The second stage of the test involves small variations in the average value of the normal force, except for during the disturbance at the end of the test. These small variations indicate that the average displacement of the pin is zero in the normal direction of contact, thus the load bearing varies very slightly, and the sliding accommodation involves very small internal third body flows. This observation is fairly consistent with the low wear rate of M1. The steady increase in friction during this period signifies the slow evolution of the rheology of the third body layer, which is involved in sliding accommodation. The friction disturbances at the test’s end are the result of material detachments causing disturbing contact openings in the interface and its third body layer. The reduction of a few micrometers in the pin position can be interpreted by the progressive loss in the third body layer, followed by its reconstitution. The reconstitution is enabled by the third bodies detached from the contact and recirculated by the rotation of the disc. In this scenario of load bearing disruption, the quantity of material lost as a result of the contact remains very small, since the pin recovers its position.

The quasi-periodic fluctuation in the normal force (Figure 12) corresponds to a cyclic disturbance of the compression of the elastic loading device, induced by the waviness of the disc friction track. Thus, these fluctuations are indicative of the pin’s normal displacement in the normal direction of contact acting as a profilometric reading of the disc’s revolutions. To facilitate this interpretation, Figure 13 presents a measurement of this apparent waviness of the disc track, deduced from the measurement of the normal force from which its average value is subtracted (high-pass filtering). The curve fluctuation appears periodic, with a period of a few seconds, which corresponds to 50 revolutions of the disc. This is due to a difference in the rotation frequency of the disc and the acquisition frequency of the force measurement. This periodicity is found in the fluctuations of the normal and tangential force measurements. A detailed analysis shows that the normal and tangential forces evolve in phase when the contact load bearing is well established, while they are out of phase during periods of accommodation and load-bearing perturbation. This is indicative of vibratory excitations occurring during the transient stage of the test. These phases are therefore visible on the friction curve over time (Figure 11a): the phase shift of the normal and tangential components of the force induces an increase in the friction fluctuation amplitude. This is clearly visible at the beginning of the test and during the disturbance of the friction at the end of the test. Finally, we note that, apart from the vibratory disturbances, the amplitude of the waviness is about 50 µm. The shape of the waviness is well reproducible from one period to the next. It corresponds to the undulation of a track with one dip and one bump per revolution, in accordance with the run-out measurements made before the tests. The oscillation amplitude of the friction track can be affected by disc wear [16,17] and third body accumulation on the friction track [25]. 

Thus, apart from the vibration-induced alterations, a variation in the amplitude of the friction track waviness is indicative of third body accumulation and circulation on the friction track, i.e., the existence of significant third body flows within the tribological circuit. 

Thus, between 80 s and 100 s, the amplitude of the apparent undulation of the friction track varies little. This period corresponds to the establishment of load bearing, during which the third body is trapped and redistributed into the interface. From 110 s to 200 s, while load bearing is well established in the contact, the amplitude of the undulation drops slightly. This indicates an accumulation of third bodies on the friction track, which is naturally more important in the valley of the track undulation, an area more prone to mechanical trapping than the bump of the track. During this period, the amount of third body varies little in the contact. At the end of the test, the perturbations of the tangential force are marked by a more significant decrease in the undulation amplitude, concomitantly with the fall of the position of the pin, i.e., with the loss of the third bodies via contact. The apparent undulation of the disc regains its amplitude before the end of the test, while the perturbation of the tangential force disappears and the pin regains its position from before the perturbation, i.e., there is a reconstitution of the third body layer in the contact. These observations indicate a very low balance in terms of the material loss of the tribological system. From these results, we conclude that the third body layer established on the surface of the M1 pin is a thin film, which evolves slightly over time, indicating low wear.

**Figure 14:**
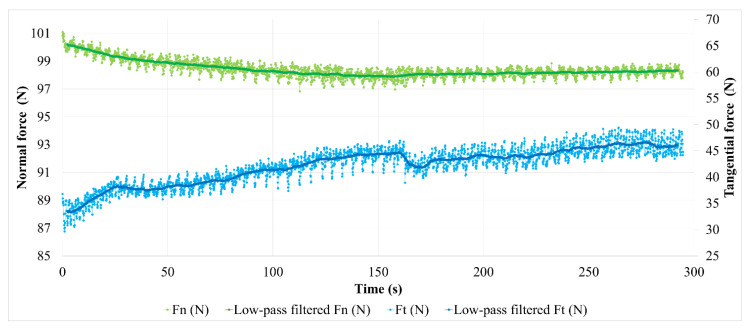
Evolution of contact forces for material M2.

Evolutions of the normal and tangential forces measured during the friction test for M2 are presented in Figure 14.

**Figure 15:**
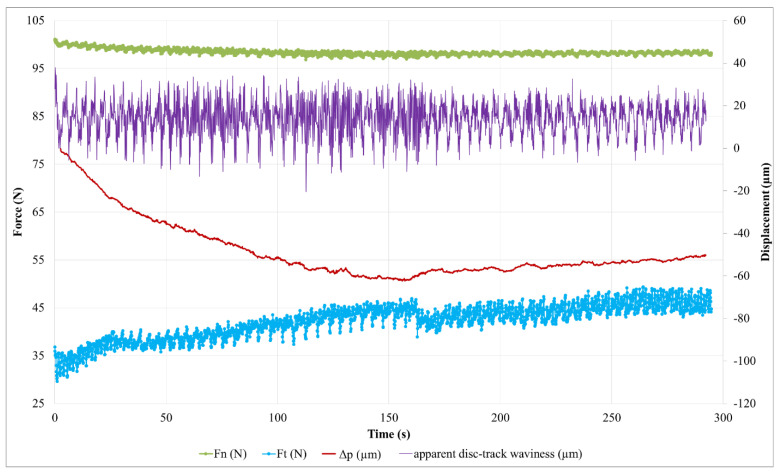
Evolution of contact forces, pin displacement (∆p) and apparent disc track waviness during the friction test for material M2.

**Figure 16:**
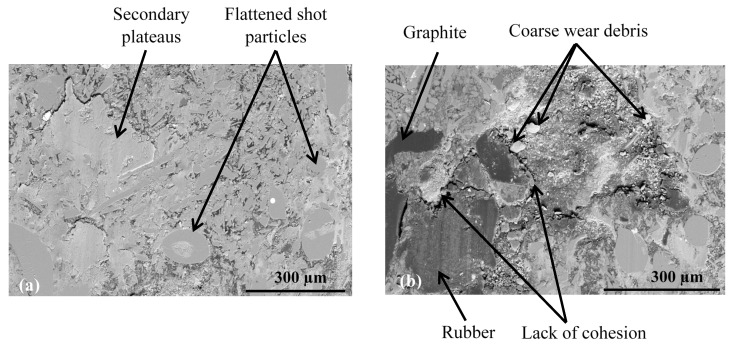
SEM observations of the third body distribution of materials (**a**) M1 and (**b**) M2 (BSE).

For the M2 material, the initial decrease in the pin’s displacement (Figure 15) indicates a transient stage of pin/disc contact accommodation of 160 s, longer than in the case of the M1 material. The longer time required to achieve compliance in the friction surfaces can be attributed to the greater difficulty of the M2 material retaining the third body in the contact. During this contact accommodation stage, the apparent waviness curve of the disc (Figure 16) is fairly disturbed, indicating significant material flows in the tribological circuit. It should be noted that the abrupt variations in friction (Figure 14) are concomitant with variations in the slope of the pin displacement (Figure 15), the latter regaining height (5 µm) from 160 s to 175 s. We attribute these variations in the pin displacement to a one-off release of the third body in the tribological circuit, then to its accumulation and redistribution in the contact. During the second part of the test, the frictional force increases more gently, similar to the M1 material (Figure 12), but in a less regular manner. In contrast to the case of the M1 material, the pin position increases steadily here (7 µm by the end of the test). This means that the tribological circuit is supplied sufficiently to allow third body accumulation in the contact. Therefore, there remains a source flow of third bodies that is attributed to the wear of the M2 material. The reduction in amplitude and the disruption of the apparent disc waviness confirm the material’s accumulation in the tribological circuit throughout this period.

From these results, we can conclude that the third body layer established on the surface of the M2 pin is unstable, which evolves significantly over time and involves high flow of third bodies. This is consistent with the higher wear here than in M1.

#### 3.4.2. Wear Behavior

EDS analyses of the third body load-bearing plateaus reveal that the weight proportions of iron provided by disc wear are similar for both pin materials, while the weight proportions of elements provided by the pin (silicon, aluminum and magnesium) are more important for M2 (Table 5). It should be noted that these chemical components derive mainly from fibers and shots of rock. Consequently, secondary load-bearing plateaus of M2 include more debris of rock fibers. The lack of cohesion revealed at the fiber/matrix interface could be the origin of the important pullout of rock fibers, and thus their great presence in the third body [38,41].

The pin wear rate (Wr) was estimated by measuring the thickness changes in the pin per unit of time, which was calculated by the following equation:

(4) $$\mathrm{Wr}=\frac{e_{2}-e_{1}}{t}$$

where e_1_ and e_2_ correspond to thickness before and after the friction test (mm), respectively, and t represents the friction test’s duration (s).

Table 6 summarizes the results of the calculated wear rates for M1 and M2. It can be seen that the wear rate is higher for M2, with 2.69 × 10^−5^ mm/s compared to 1.07 × 10^−5^ mm/s for M1. This may be related to the lack of cohesion at the matrix–rock fiber interfaces in M2. This result agrees with previous works [33,42] on the impact of the quality of the fiber–matrix interface on the wear performance of friction materials. 

According to Wan et al. [43], when the composite material has a lower compressive strength and higher fiber/matrix interfacial strength, it favors the formation of a friction layer and leads to higher and more stable friction properties. 

#### 3.4.3. Worn Surface Analysis

Macroscopic observations of the disc surface after the friction tests confirm the conclusions of the friction behavior analysis. Table 7 presents images taken of the friction track for different angular positions of the disc, in the cases of materials M1 (Table 7a) and M2 (Table 7b). In these images, the areas that appear in light gray are indicative of an abundance of third bodies present in a powdered form, compared to the darker areas where the third body layer appears essentially compacted [27]. The images show an overall heterogeneous distribution of the third body on the rubbed surface of the disc in both cases, when looking at the radial distribution. This is indicative of the location of the contact within the apparent friction area, with load bearing being established mainly in the darker areas [28]. 

In the case of M1, the dark areas, which appear to be quite extensive, are characteristic of a well-established load bearing area, with a smaller amount of powdered third body (light areas) than in the case of M2. It can also be noted that the morphology of the track varies slightly from one angular position to another, so there is little variation in the lift with the revolution of the disc in material M1. On the contrary, in the case of M2, the dark areas are more limited, and vary significantly from one angular position to another. There is hence a greater amount of powdered third body in the tribological circuit and a fluctuating bearing capacity during the revolutions. These observations are indicative of the greater wear of the M2 material compared to the M1 material.

During the contact, the brake friction materials develop millimetric plateaus of compacted third body extended in the sliding direction. They bear the load and they are known as secondary plateaus [25,28]. SEM micrographs of the worn surfaces of M1 and M2 show that the rock shots and fibers are visible, flattened, and remain embedded in the matrix (Figure 16). They contribute to the development of the load bearing surface by forming the primary support, which constitutes primary plateaus surrounded by compacted powder generating secondary plateaus (Figure 16a). Traces of third body, oriented in the sliding direction, indicate that rock fibers and shots are flattened and contribute to load bearing and speed accommodation (Figure 16). The rubber and graphite particles also remain totally or partially uncovered. However, they present traces of sliding. Consequently, they contribute to friction, but the third body does not adhere to it. Some rubber particles are partially detached, forming imprints collecting wear debris in M2 (Figure 16b). Previous studies have confirmed that rubber is the constituent most affected by the heat generated during friction, and the majority of rubber particles are prone to be partially or totally ripped and detached from the matrix [16,25]. The ability of M1 to hold rubber particles on the surface results in the better frictional stability of M1 compared to M2. In fact, rubber is an elastomer, which brings elasticity to the material, making it more compressible, and thus allowing a greater surface contact with the disc and a better distribution of surface stresses. This increases the wear resistance and improves the friction [17].

**Figure 17:**
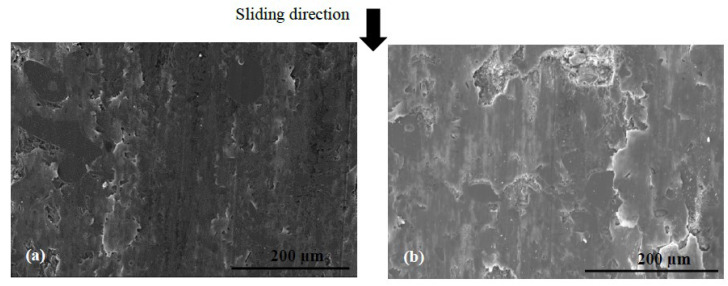
Secondary plateaus revealed in the rubbed surfaces of (**a**) M1 and (**b**) M2 (SEM, SE).

**Figure 18:**
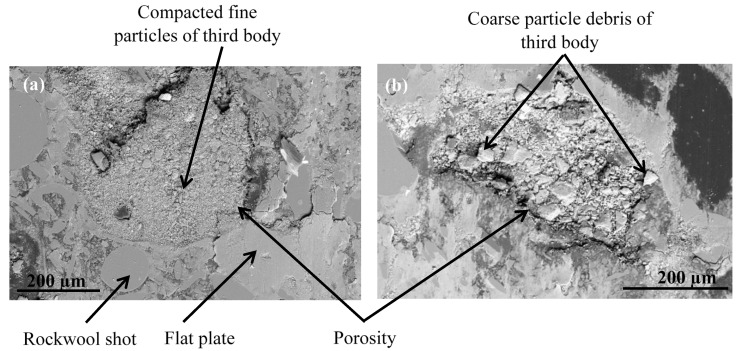
SEM micrographs of worn surfaces of (**a**) M1 and (**b**) M2 (BSE).

The SEM micrographs of the worn surface of M2 (Figure 17) display fewer secondary third body plateaus that are less extended than those of M1. Besides this, the worn surfaces of M2 reveal the presence of coarse debris, trapped in big porosities induced by fiber entanglements (Figure 16b) and the pronounced lack of cohesion in the fiber/matrix interface (Figure 16b). This debris probably results from the destruction of the secondary plateaus. All these observations indicate that M2 is less prone to develop and preserve a regular layer of third body in the contact, unlike M1. In fact, the pin surface of M1 is more covered with third body layers, which are well extended and uniformly distributed in the sliding direction (Figure 17a). The secondary load bearing plateaus are generated by third body compaction under the combined action of normal pressure and shear force, as shown in Figure 18a [44,45]. The wider flat plates revealed for M1 (Figure 17) may explain its lower wear rate, as explained by Hentati et al. [16]. Indeed, it has been proven that the formation of smooth oxide layers at the interface of rubbing materials reduced the wear rate.

In the present study, the interfacial adhesion between rock fibers and the matrix in M1 is found to be superior to that of M2. This explains the more stable friction coefficient and the higher wear resistance of M1. These results are consistent with previous works [44,46,47], which report that the improved adhesion between fibers and matrix inhibits the pullout and removal of rock fibers from the matrix during friction process. In other words, the quality of the fiber matrix interface guarantees the reinforcing efficiency of the fibers. However, a relatively weak interface bonding strength between the rock fibers and the matrix causes fiber debonding under normal pressure. Consequently, this leads to the deterioration of the stress transfer in the matrix, and affects the wear resistance performance.

Moreover, in terms of the stability of load-bearing plateaus, Figure 17a and Figure 18a confirm that the constituent distribution of M1 is more prone to form stable secondary plateaus, which further explains its better tribological behavior than M2. This result is coherent with several studies. In fact, Jara and Jang [48] showed that the formation of a regular layer of third body improves the friction stability. Horovistiz et al. [49] revealed that when secondary plateaus are homogeneously distributed over the material, as in M1, this ensures a better friction performance and provides a more homogeneous transfer film on the counterface. However, the generated third body debris increases the abrasive wear of the material, which is the case for M2 which exhibits greater wear debris (Figure 16b and Figure 18b) and a higher wear rate.

These outcomes contribute to a better understanding of the link between the microstructural characteristics and tribological behavior of composite friction materials.

From all these analyses, it can be concluded that the improved tribological behavior of M1 may be related to the superior fiber–matrix interfacial adhesion and the stable secondary third body plateaus formed on the friction surfaces, which is consistent with the previous reports on fiber-reinforced friction composites [7,47,50].

## 4. Conclusions

This paper has focused on the impact of mixing duration on the microstructure, thermo-physical and mechanical properties, and tribological behavior of organic composite friction materials. Several types of synergy between constituents were highlighted and correlated to properties, friction behavior and wear mechanisms. This study contributes to a deeper understanding and control of the link between microstructural characteristics—namely, porosity, interface defects, and constituent arrangement and distribution—and material properties and behavior. In addition, the results demonstrate the necessity of considering these materials as multi-constituent and interdependent systems, and exploring the different synergies between the constituents. It was concluded that:

1. A long mixing duration establishes a better interface between matrix and constituents, namely, rock fibers and rubber, and a more homogeneous constituent distribution, thus improving the physical (high density), thermal (low thermal expansion), mechanical (high compressive modulus) and tribological (stable friction, low wear rate) properties;
2. The large size and the arrangement of the fiber entanglements, resulting from the mixing duration, force the carbonaceous particles to concentrate. This results in dense alignments of these thermally conductive particles, which creates thermal bridges within the composite material and thus improves thermal conductivity;
3. Porosities, which result from the poor cohesion between the fiber entanglements and the matrix, have a large size and an irregular distribution over the friction surface. They generate high strain localizations and cause an irregular supply of third bodies in the tribological circuit, which disrupts the load bearing mechanisms. These porosities result in low wear resistance and disturb the frictional stability.

## Figures and Tables

**Table 1:** Formulation and constituent size of simplified materials.

Class	Constituent	Weight (%)	Mesh Opening Size (µm)
Binder	Phenolic resin	15.3	-
Filler	Calcium carbonate	21.3	-
Abrasive	Alumina	1.2	-
Lubricant	Graphite	11.9	212–300
Friction modifier	Rubber	11.3	325–400
Fiber	Rock fibers	39.1	Fibers < 400

**Table 2:** Mixing parameters of simplified material constituents.

		Material M1	Material M2
Sequences	Blended Constituents	Duration (s)	
1	50% calcium carbonate + 50% rubber + alumina	52	4
2	50% resin + 50% rubber	88	8
3	50% calcium carbonate + 50% graphite	54	4
4	50% resin + 50% graphite	52	4
5	Rock fibers	460	20
Total mixing duration		706	40

**Table 3:** Thermo-physical properties of M1 and M2.

	M1	M2
Density (kg·m^−3^)	1980 (2)	1950 (3)
Porosity (Vol%)	13.1	14.2
Thermal Conductivity λ (W·m^−1^·K^−1^)	1.18 (0.03)	1.37 (0.04)
Capacité thermique massique Cp (J·kg^−1^·K^−1^)	847 (2)	851 (5)
Thermal effusivity ε (J·K^−1^·s^−1/2^ m^−2^)	1410	1520
Coefficient of thermal expansion in normal direction α (10^−6^K^−1^)	18.7 (0.1)	19.8 (0.1)

**Table 4:** Compressive moduli of M1 and M2 materials determined for different stress levels.

	Compressive Modulus (MPa)	
Stress Levels	M1	M2
2 MPa	5515	2950
5 MPa	5645	3465
10 MPa	6100	5385

**Table 5:** Chemical composition of the third bodies in load-bearing plateaus of the M1 and M2 friction tests (wt. %).

	C	O	Si	Al	Mg	Fe
M1	13.69	28.48	8.71	4.04	1.14	31.36
M2	14	25.66	12.9	6.01	1.93	30.66

**Table 6:** Pin wear indicator (thickness loss per unit time) calculated for M1 and M2.

Materials	Wear Rate (mm/s)
M1	1.07 × 10^−5^ mm/s
M2	2.69 × 10^−5^ mm/s

**Table 7:** Images of the rubbed disc surfaces for (**a**) M1 and (**b**) M2 taken at different angular positions.

**a**	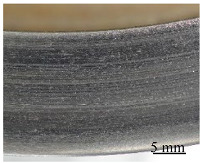	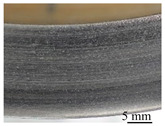	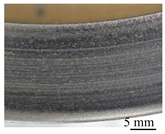
**b**	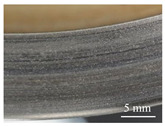	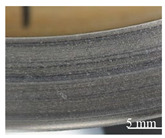	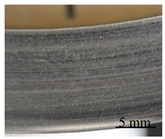
	position 1	position 2	position 3

## Data Availability

The data presented in this study are available on request from the corresponding author.
